# Nasopharyngeal carcinoma metastasis to the mammary gland: A case report

**DOI:** 10.3892/ol.2014.2656

**Published:** 2014-11-03

**Authors:** SHUANG LI, JIYUAN YANG

**Affiliations:** Department of Oncology, The First People’s Hospital of Jingzhou, Jingzhou, Hubei 434000, P.R. China

**Keywords:** nasopharyngeal carcinoma, secondary breast cancer

## Abstract

Nasopharyngeal carcinoma is the second most common type of malignancy in Southern China. Metastatic sites are usually multifocal and involve the bones, lungs and distant lymph nodes. To date, there have been no studies with regard to nasopharyngeal carcinoma metastasis to the mammary gland. In the current study, the case of a 56-year-old female with nasal obstruction, epitaxis and a bilateral neck mass is presented. Following a series of examinations, the patient was diagnosed with nasopharyngeal carcinoma (cT3N3M0). Subsequently, the patient received radical radiation therapy. After three months, a mass was identified in the left breast, together with enlargement of multiple lymph nodes in the left axilla. The patient underwent a mastectomy and pathological examination revealed that the breast mass and axillary lymph node tissues were derived from the nasopharynx. To the best of our knowledge, this is the first report of a nasopharyngeal carcinoma that metastasized to the mammary gland.

## Introduction

Nasopharyngeal carcinoma is the second most common type of malignancy in Southern China. An estimated 41,503 new cases and 20,058 mortalities were attributed to nasopharyngeal carcinoma in China in 2010, accounting for 1.34% of all new cancer cases and 1.03% of all cancer-related deaths that year in China. The incidence and mortality rates were higher among males than among females and marginally higher in urban areas than in rural areas. Among seven Chinese administrative regions, nasopharyngeal carcinoma incidence and mortality were markedly higher in South China than in other regions and lowest in North China (1). Distant metastasis, including synchronous distant metastasis at diagnosis and metachronous distant metastasis after radical radiotherapy, is a leading cause of mortality in patients with nasopharyngeal carcinoma. The lungs, bone and distant lymph nodes are the most frequent sites of distant metastases (2). Metastatic sites are usually multifocal and involve the bones, lungs and distant lymph nodes. For patients without distant metastatic lesions, radiotherapy is the standard treatment for nasopharyngeal carcinoma. To the best of our knowledge, no cases have yet been reported regarding nasopharyngeal carcinoma metastasis to the mammary gland. Written informed consent was obtained from the patient’s family.

## Case report

A 56-year-old female presented to The First People’s Hospital of Jingzhou (Hubei, China) with a two-month history of nasal obstruction, epistaxis, headaches, left-sided hearing loss and bilateral neck lymph node enlargement. Ultrasonography revealed bilateral enlargement of the cervical lymph nodes (diameter, 3–5 cm). On nasopharyngoscopy, a mass in the nasopharynx was identified and biopsied. Histopathological examination revealed a non-keratinizing carcinoma of the nasopharynx (World Health Organization type III) (3). Computed tomography (CT) and magnetic resonance imaging (MRI) revealed extension of the tumor to the nasal fossa and the base of the skull (T3), as well as to the lymph nodes of the left supraclavicular fossa (N3). No signs of lung or liver metastases were identified on CT scans and a bone scan showed negative results (M0). According to the American Joint Committee on Cancer Staging (4), the patient was diagnosed with stage IV nasopharyngeal carcinoma.

The patient refused induction chemotherapy and concurrent chemoradiation therapy, and underwent radiation therapy for two months, whereby a total dose of 76 Gy (2 Gy per fraction, total 38 fractions) was delivered to the primary tumor and 60 Gy (2 Gy per fraction, total 30 fractions) to the bilateral neck. Total locoregional control was achieved. The patient refused additional treatment.

After three months, a mass was identified in the left breast. No signs of lung, liver or bone metastases were identified on CT scan and MRI. The patient underwent a mastectomy, and during the surgery, a mass with a diameter of 4x3x3 cm was identified in the outer upper quadrant, together with enlargement of multiple lymph nodes in the left axilla.

**Figure 1:**
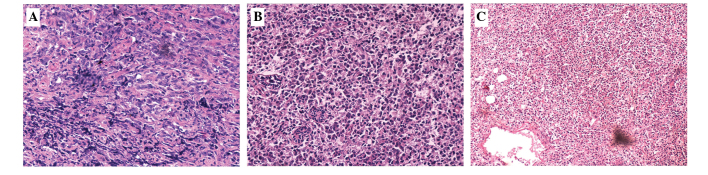
Immunohistochemical staining of the tissues was performed. (A) Nasopharyngeal carcinoma with dark nucleoli (hematoxylin and eosin staining; magification, x200). (B and C) Tumor tissue identified in the breast. (B) Hematoxylin and eosin staining; magnification, x200. (C) Hematoxylin and eosin staining; magnification, x100.

**Figure 2:**
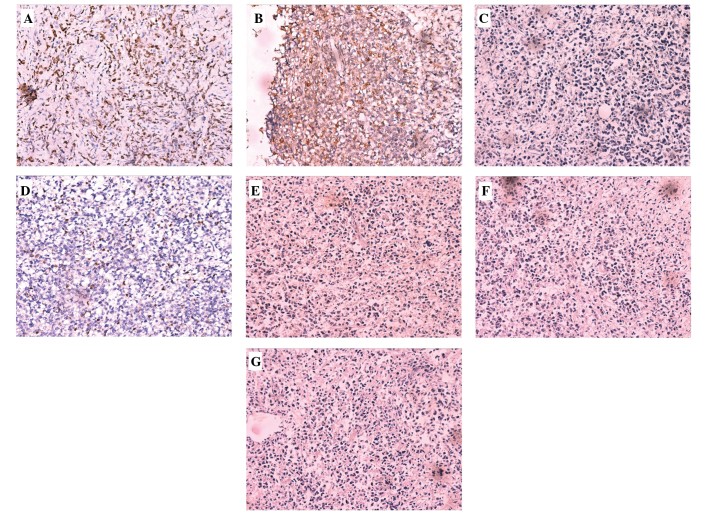
Immunohistochemical staining of (A) nasopharyngeal carcinoma and (B–G) breast tumor. The two tissues were positive for (A and B) CK17. The breast tumor tissue exhibited negative staining for (C) CK20, (D) CD3, (E) CD20, (F) synaptophysin and (G) chromogranin A (magnification, x200). CK, cytokeratin; CD, cluster of differentiation.

Histological examination revealed left axillary lymph node metastasis and a malignant tumor in the left breast (non-keratinizing carcinoma), similar to the nasopharyngeal carcinoma. Immunohistological examination of the tumor tissue revealed positivity for pan cytokeratin (PCK) and CK17, and negativity for CK20, cluster of differentiation (CD)3, CD20, chromogranin A and synaptophysin (Figs. 1 and 2). In normal breast tissue, CD3, CD20, chromogranin A and synaptophysin are all negative under immunohistological examination. The patient was diagnosed with nasopharyngeal carcinoma, with metastasis to the left breast and left axillary lymph nodes (rT0N0M1). The patient refused any further treatment and succumbed to multiple organ failure after six months.

## Discussion

Malignant neoplasms rarely metastasize to the mammary gland, the incidence of which is reported to be only 0.5–2%, worldwide (5–8). Such neoplasms include malignant lymphoma, malignant melanoma, lung cancer, gastric cancer, prostate carcinoma and ovarian cancer (9). However, there have been no studies with regard to the metastasis of nasopharyngeal cancer to the mammary gland. In the present case, the patient was firstly diagnosed with advanced nasopharyngeal carcinoma, however, a mass was then identified in the breast and the pathological and immunohistochemistry results indicated that this was derived from the nasopharynx. This indicates that nasopharyngeal cancer may metastasize to the breast and the axillary lymph nodes.

The mechanism by which nasopharyngeal carcinoma metastasizes to the axillary lymph nodes may be by retrograde lymphatic drainage due to obstruction (10). In the present case, the patient presented with enlargement of the left supraclavicular lymph nodes; when the large lymph nodes block the lymphatic ducts, metastasis can spread retrograde to the axillary lymph nodes.

Secondary breast malignancies exhibit an extremely poor prognosis, even when excluding metastatic spread from the contralateral breast, which has a markedly lower prognosis than primary breast cancer. The most common sites of distant metastases of nasopharyngeal carcinoma are bone, the lungs, liver and distant lymph nodes (11). The median survival time following diagnosis of distant metastasis is extremely variable depending on the site, and the disease-free survival rate has been reported to range between 82 and 190 months (12). Combined treatment with systemic chemotherapy and locoregional radiotherapy or lumpectomy is recommended, as mastectomy may not have a benefit on overall survival.

In conclusion, the present study is the first reported case of nasopharyngeal carcinoma with metastasis to the mammary gland. When breast tumors are identified in patients with a past history of malignant disease, the possibility of breast metastasis must be considered. Secondary breast malignancy indicates a poor prognosis (8). It is important to avoid unnecessary radical surgery and to perform the appropriate systemic therapy.
